# A Novel Combined Mung Bean and Mulberry Powder: Combination Index and Shelf Life of Total Phenolic, Anthocyanin, and GABA Contents and Neuroprotective Activity

**DOI:** 10.3390/foods14060993

**Published:** 2025-03-14

**Authors:** Pontapan Polyiam, Jintanaporn Wattanathorn, Wipawee Thukhammee

**Affiliations:** 1 Neuroscience Program, Department of Physiology and Graduate School, Faculty of Medicine, Khon Kaen University, Khon Kaen 40002, Thailand; pontpo@kku.ac.th; 2 Research Institute for Human High Performance and Health Promotion (HHP&HP), Khon Kaen University, Khon Kaen 40002, Thailand; jinwat05@gmail.com; 3 Department of Physiology, Faculty of Medicine, Khon Kaen University, Khon Kaen 40002, Thailand

**Keywords:** mung bean, mulberry fruit, neuroprotective effects, natural products, plant-based products, shelf life

## Abstract

Plant-based products are widely used in the food industry. This study aims to develop neurofunctional ingredients derived from mung beans with mulberry fruit powder, evaluate their phytochemical contents (total phenolic, anthocyanin, and GABA contents), impact of anti-oxidant activities (DPPH free radical scavenging inhibition and Ferric Reducing Ability Power (FRAP)) and neuroprotective activities (acetylcholinesterase (AChE), monoamine oxidase (MAO), MAO type A, and gamma-aminobutyric acid transaminase (GABA-T)), and focus on their shelf life. Result: A total of nine ratios of mung beans and mulberry fruit powder mix were evaluated, which showed that a ratio of 1:3 (g/g) provided better IC50 values of antioxidant and neuroprotective activities than other ratios, and showed a combination index (CI < 1) which was interpreted as a synergistic effect on AChE inhibition. Thus, this ratio was selected to make freeze-dried powder (mung bean mix mulberry fruit powder (MMP)), and its shelf life was evaluated as showing stability in its phytochemical contents (except GABA, which was reduced by more than 50% at 30 ± 2 °C) and antioxidant and neuroprotective activities, which remained stabilized at more than 50% in both real-time and accelerated conditions for 6 months and 8 weeks, respectively. During 1 to 6 months of storage at 4 °C, IC50 values of FRAP showed inhibited DPPH, AChE, MAO, MAO-A, and GABA-T levels in ranges of 4.43–6.69 mg/mL, 4.10–4.68 mg/mL, 5.18–5.90 mg/mL, 4.95–5.43 mg/mL, 5.93–6.42 mg/mL, and 5.05–5.53 mg/mL respectively, not significantly different when compared to 0 months. Conclusion: These findings indicate that the shelf life of the bioactivities of MMP remain stabilized for up to six months, so it could be applied in the food industry for use as a healthy plant-based supplement.

## 1. Introduction

Stress, anxiety, and depression are among the major public global health problems. The Global Health Data Exchange has reported that about 301 million people in the world have an anxiety disorder, which is the most common mental disorder [1]. In 2020, the estimated number of people living with anxiety and depressive disorders rose 26% and 28% percent, respectively, in one year, primarily due to the COVID-19 pandemic [2,3]. Moreover, chronic diseases (including diabetes, heart disease, cancer, respiratory problems, etc.) are among the factors associated with triggering stress, anxiety, and depression [4].

Recently, a review has discussed the evidence indicating that the human gut is directly linked to the brain and central nervous system (CNS) in a system called the gut–brain axis, which affects mental health status (such as stress and anxiety changes) as well as immune function in humans [5,6]. The effects of stress and diet on the “Brain–Gut” and “Gut–Brain” pathways are linked to the impacts of diet on stress and depression changes in animal models [7]. The review found that the effect of *Lactobacillus rhamnosus* could reduce and prevent the production of cortisone during stress induction, and it also found that *Lactobacillus* and *Bifidobacterium* can influence regulation of the Hypothalamic–Pituitary–Adrenal (HPA) axis related to stress states [8]. Dietary fiber can be fermented by gut microbiota and produce short-chain fatty acids (SCFAs) such as butyrate, acetate, and propionate, which act as neuroactive compounds in the blood and can play a role in the brain through two kinds of transmembrane G protein-coupled receptors or free fatty acid receptors [9].

People are interested in plant-based foods due to the health benefits of their pharmacological properties and neuroprotective activities, which have previously been described. Plant-based proteins concentrated from mulberry leaves and mung beans contain phytochemical compounds, e.g., phenolic and flavonoid content, and contain antioxidants, anticholinesterase, monoamine oxidase, and γ-aminobutyric acid transaminase [10]. Food nutrients can affect concentrations of γ-aminobutyric acid (GABA) and stress hormones (cortisol). GABA reduces the secretion of cortisol by decreasing the release of corticotropin-releasing hormone (CRH) [11]. Tryptophan is an essential amino acid which is a precursor to the monoaminergic neurotransmitter serotonin and may lead to a reduction in cortisol levels [11]. It is found in beans such as mung bean protein isolate at around 6.4 mg/g [12]. Mung beans have been reported to reduce stress symptoms due to the properties of GABA. Interestingly, a previous study compared the anti-stress effects of bean fermentation and germinate extracts, and the results indicated that mung bean fermentation always showed better anti-stress and antioxidant properties than germinated mung beans [13]. Studies have shown that a fermented product of soybeans, black beans, and green bean mixture is a potential anticancer nutritional supplement [14].

Mulberry fruits have bioactive compounds such as anthocyanins, rutin, and quercetin that show antioxidant, neuroprotective, and immunomodulation properties in both in vitro and in vivo trials [15]. Moreover, studies of their effects in animals have found that increased cholinergic function and neuroprotective activity occur partly due to decreased oxidative stress and neuronal apoptosis [16]. A human study reported that children aged between six and twelve years old who received milk containing mulberry extract had decreased levels of saliva cortisol, a stress hormone [17]. Interestingly, mung beans are an excellent source of protein and combined with mulberry fruit as a powerful antioxidant and neuroprotector, provides powerful raw materials that can develop as a combination to promote health. However, there are still a lack of data to support their combination index on phytochemical contents, stability of bioactivities, shelf life, physicochemical characteristics, and nutritional contents to provide a functional ingredient to modulate neuroprotective parameters. Furthermore, clearly the coordination between mung bean milk and mulberries can also combine with other plant source ingredients for use in targeting applications.

Therefore, this study aims to evaluate the combination index of different ratios of mung bean milk and mulberry fruit to develop the neurofunctional ingredients of a freeze-dried powder-derived mung bean and mulberry fruit mixture and evaluate its phytochemical contents (total phenolic, anthocyanin, and GABA contents), antioxidant activities (DPPH free radical scavenging inhibition and the Ferric Reducing Ability Power (FRAP)), neuroprotective activities (acetylcholinesterase (AChE), monoamine oxidase (MAO), MAO type A, and gamma-aminobutyric acid transaminase (GABA-T)), their shelf lives, and nutritional facts.

## 2. Materials and Methods

### 2.1. The Plant Material

**Figure 1:**
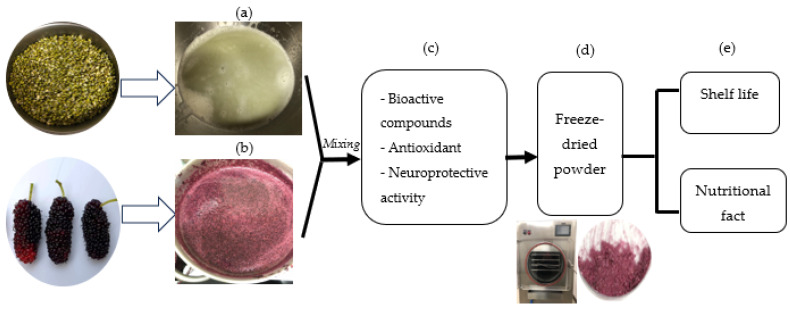
Plant material and research process. (**a**) Mung bean milk; (**b**) mulberry fruit; (**c**) evaluated total phytochemical contents (total phenolic, anthocyanin, and GABA contents), antioxidant activities (DPPH free radical scavenging inhibition and the Ferric Reducing Ability Power (FRAP)), and neuroprotective activities (acetylcholinesterase (AChE), monoamine oxidase (MAO), MAO type A, and gamma-aminobutyric acid transaminase (GABA-T)); (**d**) freeze-drying process and; (**e**) shelf life and nutritional facts.

Mulberry fruits, or *Morus alba* L., of the Chiang Mai 60 species, were cultivated and harvested between 2022 and 2023 in Udon Thani Province, Thailand. Mung bean seeds (Raitip^®^ brand, Thanya Farm Co., Ltd. Bangyai, Nonthaburi, Thailand) were purchased from a supermarket in Khon Kaen province, Thailand. In summary, a research process as shown in Figure 1.

### 2.2. Sample Preparation to Select the Functional Ingredient

Mung beans were rinsed with tap water three times and then soaked in drinking water (1:5 ratio) overnight. After that, the beans were mixed with drinking water in a blender. The finely blended beans were filtrated with a grid to remove debris. The mung bean milk was then heated and stirred at about 95 °C for 10 min, before resting it until it returned to room temperature.

The combined process was adapted from a previous study [14]. Mulberry fruits (MFs) were rinsed with tap water then blended with mung bean milk in various ratios to find the ratio with the greatest synergistic effect according to combination index. The nine ratios tested were 1:1, 1:2, 1:3, 1:4, 1:5, 2:1, 3:1, 4:1, and 5:1. Nine ratio samples were contained in sterile glass bottles with closed lids and pasteurized at 70 °C for 5 min. All samples were organically fermented at room temperature (~30 °C) overnight (before fermentation, the pH value was about 6.01 ± 0.02, and after fermentation it was 4.40 ± 0.05), then each ratio was aliquoted (5 mL) to screen for their biological activities. Finally, the ratio that showed the best biological activities was chosen to receive added cryoprotection by maltodextrin (1:1 *v*/*v*), for preparation as a freeze-dried powder, and for shelf-life analysis.

Freeze-drying was conducted using a Vacuum Freeze Dryer (Grisrianthong Co., Ltd., Samutsakorn, Thailand) by the following process: The 1st step is the pre-freezing at −20 °C for 120 min; then the 2nd step is the primary drying phase, including steps 1, 2, 3, and 4 at −30 °C for 120 min, 10 °C for 390 min, 20 °C for 300 min, and 30 °C for 300 min, respectively; and the 3rd step is the secondary drying phase, including steps 1 and 2 at 30 °C for 540 min, and hold on at 30 °C for 540 min, respectively. The total duration time of freeze-drying is about 48 h or twice overnight.

### 2.3. Analysis of Phytochemical Contents by Screening Functional Ingredients

#### 2.3.1. Total Phenolic Contents

All samples in each ratio were analyzed for total phenolic contents by the modified Folin–Ciocalteu method [10,18,19]. Briefly, 20 µL of extract samples (10 mg/mL in methanol) were mixed with 120 µL of Folin–Ciocalteu reagent (50% *v*/*v*) in 96-well microtiter plates and incubated at room temperature for 8 min, avoiding the light. Then, 30 µL of 20% sodium carbonate (Na_2_CO_3_) was added and incubated at 30 ± 2 °C in the dark for 2 h. After incubation, the plate was measured with an absorbance of 765 nm by a microplate reader. Various concentrations (0.5–500 µg/mL) of gallic acid were used to prepare a standard calibration curve (y = 1.1702x + 0.0132, R^2^ = 0.997). The results were expressed as mg of gallic acid equivalent (GAE)/g of sample in triplicate experiments.

#### 2.3.2. Total Anthocyanin Content

Anthocyanins are a type of pigment found in fruits. Measurement of anthocyanin content was carried out by the pH differential method [20]. In brief, the mixture of buffers (150 µL of pH 1.0 buffer (0.025 M potassium chloride) and 150 µL of pH 4.5 buffer (0.4 M sodium acetate)) was mixed with 50 µL of the sample. Then, a color change from pink-red to blue-violet with a change in pH value from 1.0 to 4.5 indicates the presence of anthocyanin and the absorbance values at 520 and 700 nm is measured with a microplate reader. The results were reported as mg Cyanidin-3-Glucoside (C3G)/g of sample in triplicate experiments. Total anthocyanin content (TAC) was calculated by the following equation:

TAC = (A × MW × DF × 10^3^)/ε

where

A = (A520 − A700) pH 1.0 − (A520 − A700) pH 4.5,

MW = 449.2 g/moL for cyanidin-3-glucoside,

DF = Dilution factor (10),

10^3^ = Factor for conversion from g to mg,

ε = The molar extinction coefficient for 26,900 L/mol/cm.

#### 2.3.3. Total GABA Content

The total gamma-aminobutyric acid (GABA) content was evaluated by a process adapted from that previously described in [21]. Briefly, one gram of the sample was added to 5 mL of distilled water and centrifuged at 4000 rpm for 60 min. Then, 0.5 mL of the supernatant were pipetted and 0.2 mL of borate buffer solution (pH 9), 1 mL of 6% phenol solution, and 0.4 mL of sodium hypochlorite solution were added and shaken to mix. Then, the mixture was boiled in a water bath for 10 min and removed and immersed in a cold-water bath for 20 min. Then, 2 mL of 60% ethanol was added. The solution obtained was measured for absorbance at 645 nm with a UV-VIS spectrophotometer. GABA (Sigma-Aldrich Co. LLC, St. Louis, MO, USA) was used as the standard curve (y = 2.115x − 0.0035, R^2^ = 0.999). The results were expressed in mg/g of sample in triplicate experiments.

### 2.4. Analysis of Antioxidant Activity

#### 2.4.1. DPPH (1,1-Diphenyl-2-picrylhydrazyl Radical) Inhibition

The DPPH radical scavenging method was carried out as previously described [10,22]. In brief, DPPH solution was aliquoted in methanol and mixed with 20 μL of the sample at various concentrations from 5 to 1000 μg/mL, then was incubated at room temperature, avoiding light, for 30 min. After incubation, the sample was measured with a microplate reader at an absorbance of 517 nm. The results were revealed as the percentage inhibition of DPPH radical formation, while L-ascorbic acid (μg/mL) (Sigma-Aldrich, St. Louis, MO, USA) was used as the standard curve (y = 0.5066x + 35.902, R^2^ = 0.990) to calculate the half maximal inhibitory concentration (IC_50_) in mg per mL. The samples were assessed in triplicate.

#### 2.4.2. FRAP (Ferric Reducing Ability Power)

The FRAP assay was carried out as previously published [19]. The FRAP assay was performed by determining the capacity of the reducing substance from ferric tripyridyl triazine (Fe^3+^-TPTZ) to convert ferrous tripyridyl triazine (Fe^2+^-TPTZ). The FRAP working solution was prepared with a mixture of 300 mM of acetate buffer, 10 mM of TPTZ, and 20 mM of ferric chloride (FeCl_3_) solutions at a ratio of 10:1:1, respectively. Briefly, 190 µL of FRAP reagent was added with 10 µL of the sample and incubated at 37 °C for 10 min. After that, the mixture was determined by absorbance at 593 nm by a microplate reader. Trolox was used as the standard curve (y = 0.0014x − 0.0038, R^2^ = 0.997) to calculate the IC_50_. The results were reported as IC_50_ values in mg per mL in triplicate experiments.

### 2.5. Analysis of Neurotransmitter Inhibition Activity

#### 2.5.1. AChE (Acetylcholinesterase Enzyme) Inhibition

The AChE suppression activity of each extract was determined using the colorimetric method previously described in [10,19]. A rat cerebral cortex tissue sample was isolated and prepared as a homogenate in 0.1 M of potassium phosphate buffer (pH 7.4) (1:5 *w*/*v*) and subjected to 12,000 rpm centrifugation at 4 °C for 10 min. The supernatant was harvested and used as a source of AChE. Briefly, 25 µL samples at various concentrations were incubated with the reaction mixture containing 25 µL of 15 mM ATCI (acetylthiocholine iodide) (Sigma-Aldrich, St. Louis, MO, USA), 75 µL of 3 mM DTNB (5,5′-dithio-bis-2-nitrobenzoic acid) (Sigma-Aldrich, St. Louis, MO, USA), and 50 µL of 50 mM Tris-HCl (pH 8.0) at room temperature for 5 min. After mixing, the absorbance at 412 nm was recorded with a microplate reader both before and after adding 0.22 U/mL of AChE (25 µL). The percentage of inhibition was calculated by comparison between the rate of hydrolysis of the ATCI in the samples and that of the blank (Tris-buffer). The results are shown as the percentage inhibition of AChE and the IC_50_ value of each sample. Donepezil (ARICEPT^®^, New York, NY, USA) was used as a reference standard. Each sample was assessed in triplicate.

#### 2.5.2. MAO (Monoamine Oxidase Enzyme) Inhibition

MAO is a key enzyme in the degradation of a wide range of endogenous monoamine neurotransmitters, such as noradrenaline, dopamine, and serotonin. MAO inhibition activity was measured by using the colorimetric method previously described [10]. A chromogenic solution was prepared for inclusion in the assay mixture containing 1 mM of vanillic acid, 500 µM of 4-aminoantipyrine, and peroxidase (4 U/mL) in potassium phosphate buffer (0.2 M, pH 7.6). The source of MAO was prepared from the rat cerebral cortex tissue, as described above. The assay mixture contained 25 µL of monoamine oxidase, 25 µL of various concentrations each of the sample, 50 µL of chromogenic solution, and 200 µL of 500 µM P-Tyramine. After mixing, the reaction mixture was incubated at 37 °C for 30 min and the absorbance at 490 nm was recorded with a microplate reader. The results are shown as the percentage inhibition of MAO and the IC_50_ value of each sample. Hydrogen peroxide (H_2_O_2_, AR grade, QRëC™, ASIA CHEMIE (Thailand) Co., Ltd., Chonburi, Thailand.) was used as a reference standard. Each sample was assessed in triplicate.

#### 2.5.3. MAO-A (Monoamine Oxidase Enzyme Type-A) Inhibition

MAO-A inhibitors are efficacious for treating anxiety and depression [23]. Monoamine oxidase (MAO) type A activities were determined by using the colorimetric method with a microplate reader [10]. The reagents and the source of MAO-A were prepared similarly to the total MAO inhibition assay described above. Briefly, the mixture contained 25 µL of monoamine oxidase, 25 µL of various concentrations of sample, 50 µL of chromogenic solution, and 50 µL of 500 nM pargyline and was incubated at 37 °C for 30 min. Then, 150 µL of 500 µM P-tyramine was added and the absorbance at 490 nm was recorded by microplate reader. The results were shown as percentage inhibition of MAO-A and the IC_50_ value of each sample. H_2_O_2_ was used as a reference standard. Each sample was assessed in triplicate.

#### 2.5.4. Gamma-Aminobutyric Acid Transaminase (GABA-T) Inhibition

GABA-T activity was assessed using an enzymatic assay, as previously described in [10]. The source of GABA in the cerebral cortex tissue of a rat was isolated and prepared as a homogenate in 0.1 M of potassium phosphate buffer (pH 7.4) (1:5 *w*/*v*) and subjected to 12,000 rpm centrifugation at 4 °C for 10 min. The supernatant was harvested and used as a source of GABA-T. The assay mixture contained 800 µL of GABA-T Buffer (20 mM of gamma-aminobutyric acid (GABA), 10 mM of Alpha-ketoglutarate, and 0.5 Mm of NAD in 0.5 M of sodium phosphate buffer (pH 8.0)), 200 µL of brain supernatant, and 200 µL of plant protein sample. In the reactions, NAD+ was reduced to NADH, allowing the quantification of GABA-T through spectrophotometric measurement at 340 nm. The results are shown as percentage inhibition of GABA-T and the IC_50_ value of each sample. Vigabatrin (500 mg tablet (Sabril^®^, Paris, France)) was used as a reference standard. Each sample was assessed in triplicate.

### 2.6. Combination Index (CI) and Dose Reduction Index (DRI)

The *CI* method used was that previously described by Chou–Talalay (1984) [24], to investigate synergism or antagonism by the in vitro pharmacodynamic interactions between mung beans and mulberry fruits comparing individual components. Synergism is defined as CI < 1, summation is indicated by CI = 1, and CI > 1 indicates antagonism. This can be calculated by the following formula:

(1) $$\mathrm{CI}=\;\frac{\left(\mathrm{IC}50\;combi\right)1}{\left(\mathrm{IC}50\;alone\right)1}+\;\frac{\left(\mathrm{IC}50\;combi\right)2}{\left(\mathrm{IC}50\;alone\right)2}$$

For example,  CI_AChE_ = (1.484/4.952) + (1.484)/2.528)

                    = 0.886; Result was interpreted as a synergism

The dose reduction index (DRI) method of Chou and Chou (1988) [25] was used to investigate combination studies and their synergy quantifications using the Chou–Talalay method (1984) [24]. This can be calculated by the following formula:

(2) $$\mathrm{DRI}=\;\frac{\left(\mathrm{IC}50\;alone\right)1}{\left(\mathrm{IC}50\;combi\right)1}$$

For example,  DRI_AChE_ (mungbean alone) = 4.952/1.484 = 3.336

         DRI_AChE_ (mulberry alone) = 2.528/1.484 = 1.703

### 2.7. Shelf Life of a Mung Bean + Mulberry Fruit (1:3) Freeze-Dried Powder (MMP)

Assessment of the shelf life of MMP followed the procedure from the previous study and was separated into two condition tests, including real-time [26] and accelerated shelf-life testing [27]. After the freeze-drying procedure, the products were divided into aluminum foil bags and stored for periods of 0, 1, 3, and 6 months (at room temperature 30 ± 2 °C and 4 °C in the refrigerator) for real-time testing, while for accelerated testing the products were stored at 40 °C in an incubator (Memmert, Schwabach, Germany) for 0, 2, 4, 8 weeks. All the products (both for real-time and accelerated testing) were separated and immediately stored on the same day, without opening the door of the refrigerator/incubator until the time of the measurements. Each product was then measured for total phenolic, anthocyanin, and GABA contents, antioxidant activities (e.g., DPPH and FRAP assays), and neuroprotective activities (e.g., AChE, MAO, MAO-A, and GABA-T), which were carried out in triplicate experiments.

### 2.8. MMP Powder Experiment

#### 2.8.1. PH Value

Five grams of powder sample were dissolved with distilled water and the pH value was measured by Waterproof pH Meter (SANXIN pH5S instrumentation, Inc., Shanghai, China) in triplicate experiments.

#### 2.8.2. Moisture Content

The moisture content (%) was measured in a sample of 5 g of powder according to a method previously described in [28]. The moisture content was determined by using a moisture analyzer (Nanjing Bonita Scientific Instrument Co., Ltd., Nanjing, China) in triplicate experiments.

#### 2.8.3. Color Measurement

The *L∗*, *a∗*, and *b∗* values were determined according to a method previously described in [29] by using an FRU^®^ Precise Color Reader WR-10 (ShenZhen Wave Optoelectronics Technology Co., Ltd., Shenzhen, China) in triplicate experiments.

#### 2.8.4. Brix Measurement

The % Brix value was determined using a Digital/Optical Refractometer (Hanna Instruments, HI 96801, Woonsocket, RI, USA) in triplicate experiments. Limit range detection—0 to 85%; resolution—±0.1%; accuracy—±0.2%.

### 2.9. Nutritional Facts

A total of 1.1 kg of a freeze-dried powder of Mung bean and Mulberry fruit mix (MMP) was delivered by post for 2 days (room temperature) to ALS Laboratory Group (Thailand) Co., Ltd. (Bangkok, Thailand). The nutritional content was analyzed according to the Nutrition Labeling guidelines (1993), JAOAC (1993), AOAC (2019), and various AOAC methods (2023), as reported by the ALS Labor-atory Group (Bangkok, Thailand).

### 2.10. Statistical Analysis

All experimental data were represented as mean ± standard error of the mean (SEM). All data were compared between samples by one-way ANOVA analysis and Tukey’s HSD test (a—*p*-value < 0.05 was accepted as significant) for all conditions. Statistical analyses were conducted using the computer software SPSS 21.0 (IBM SPSS Statistics for Windows).

## 3. Results

### 3.1. Biological Activities After Screening the Nine Ratios of Mung Bean/Mulberry for Antioxidant and Neuroprotective Activities

Previous evidence has indicated that plants that show acetylcholinesterase inhibitor (AChEI) activity could promote neuroprotection [10,30]. MAO plays a role in the breakdown of monoamine transmitters (e.g., serotonin, dopamine, and adrenaline) involved in mental health status as well as targeting drugs by inhibition of MAO [31]. GABA is a major inhibitory neurotransmitter that plays a crucial role in calming the human nervous system and it is rapidly changed to succinate and glutamate by GABA transaminase (GABA-T) [32]. Neurobiological parameters that effect neurotransmitter changes include AChE inhibition [30], MAO inhibition, MAO-A inhibition [31], and GABA-T inhibition [32]. From screening the nine ratios of mung bean/mulberry, the results revealed that mung bean/mulberry at a ratio of 1:3 (g/g) showed better IC50 values of antioxidants (e.g., DPPH inhibition and FRAP) and neuroprotective activities (e.g., AChE, MAO, MAO-A, and GABA-T) than other ratios, as shown in Table 1.

### 3.2. The Combination Index (CI) and Dose Reduction Index (DRI) of a Mung Bean + Mulberry Fruit Mix (1:3)

The combination index (CI) of mung bean/mulberry at a ratio 1:3 (g/g) showed the synergistic effect of neuroprotection on AChE inhibition and inhibition of MAO, MAO-A, and GABA-T, which were interpreted as additive effects, while antioxidant (DPPH inhibition and FRAP) effects but not synergistic effects are shown in Table 2.

**Figure 2:**
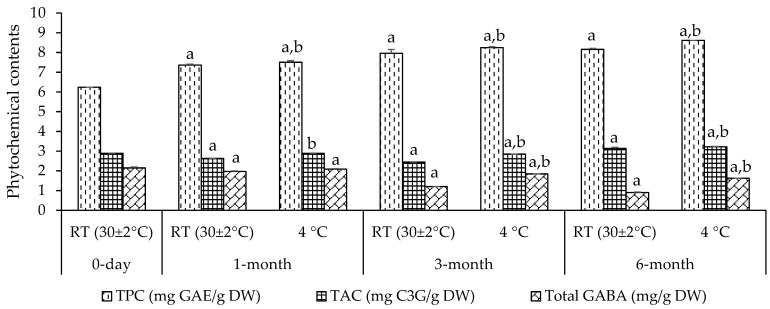
Real-Time shelf-life stability of phytochemical contents (total phenolic content, total anthocyanin content, and total gamma-aminobutyric acid) of an MMP at room temperature and 4 °C for 0, 1, 3, and 6 months. Data are presented as mean ± SEM (in triplicate experiments). TPC—total phenolic content—TAC—total anthocyanin content; GABA—gamma-aminobutyric acid; C3G—cyanidin-3-glucoside; GAE—gallic acid equivalents; DW—dry weight; MMP—mung bean + mulberry fruit (1:3) freeze-dried powder; RT—room temperature; ^a, b^—*p* < 0.05 when compared at 0-day and at RT each time, respectively, Statistical test by Tukey’s HSD test.

After we found that the best ratio of mung bean + mulberry (1:3) provides the highest bioactivity, it was developed as a freeze-dried powder (MMP). Real-time stability tests of MMP indicate that the level of total phenolic content (TPC), total anthocyanin content (TAC), and total gamma-aminobutyric acid (GABA) content remain stable for up to 6 months at both room temperature (30 ± 2 °C) and 4 °C (except GABA at room temperature) as shown in Figure 2.

**Figure 3:**
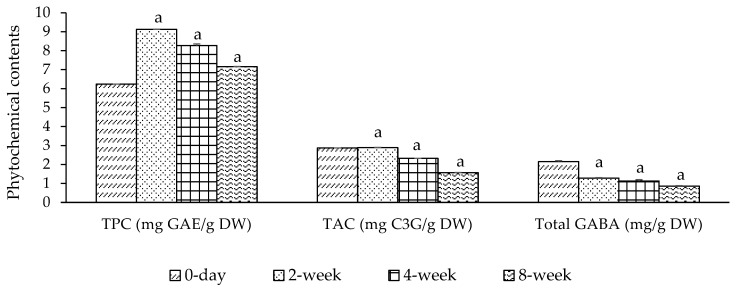
Accelerated shelf life of phytochemical contents (total phenolic content, total anthocyanin content, and total gamma-aminobutyric acid) of an MMP at 40 °C for 0, 2, 4, and 8 weeks. Data are presented as mean ± SEM (in triplicate experiments). TPC—total phenolic content; TAC—total anthocyanin content; GABA—gamma-aminobutyric acid; C3G—cyanidin-3-glucoside; GAE—gallic acid equivalents; DW—dry weight; MMP—mung beans + mulberry fruit (1:3) freeze-dried powder; ^a^—*p* < 0.05 when compared at 0-day. Statistical test by Tukey’s HSD test.

We found that the accelerated shelf life of mung bean + mulberry powder (MMP) shows the level of total phenolic content (TPC), and total anthocyanin content (TAC) from 0 days to 2 weeks and these contents increased from 6.239 to 9.127 mg GAE/g, and 2.869 to 2.890 mg C3G/g, respectively, which may be caused by loss of moisture content. After that, these phytochemical contents sequentially reduced to 7.161 mg GAE/g and 1.570 mg C3G/g, respectively, while total gamma-aminobutyric acid (GABA) content from 2 weeks to 8 weeks sequentially reduced from 2.151 mg/g to 0.859 mg/g, indicating that temperature is the major factor in reducing shelf life, as shown in Figure 3.

### 3.3. Shelf Life of the Biological Activities of a Mung Bean + Mulberry Fruit (1:3) Freeze-Dried Powder (MMP)

The results for the real-time stability of the antioxidative and neuroprotective activities of MMP remained stabilized. The bioactivities were prolonged to 6 months at both temperatures when compared with 0-day (except inhibition of DPPH and decreasing FRAP (IC50 value raised), which were significantly different after 3 months of storage). It was found that a temperature difference of 4 °C showed IC50 values of FRAP and inhibition of DPPH, AChE, and MAO-A significantly better than room temperature (30 ± 2 °C) after storage for 1, 3, and 6 months, and GABA-T inhibition differed at 3 and 6 months, while MAO inhibition was not different at either temperature until 6 months. Thus, high temperature is a factor that affects bioactivities, as shown in Table 3.

**Figure 4:**
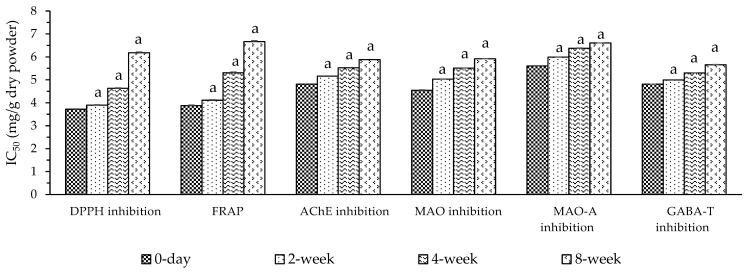
Accelerated shelf life of the antioxidative and neuroprotective activities of mung bean + mulberry fruit powder (MMP) at 40 °C for 0, 2, 4, and 8 weeks. Data are presented as mean ± SEM (in triplicate experiments). DPPH—1,1-diphenyl-2-picryl hydrazyl radicals; FRAP—Ferric Reducing Ability Power; AChE—acetylcholinesterase; MAO—monoamine oxidase; MAO-A—monoamine oxidase type A; GABA-T—gamma-aminobutyric acid transaminase; MMP—mung bean + mulberry fruit (1:3) freeze-dried powder; IC_50_—half maximal inhibitory concentration. ^a^—*p* < 0.05 when compared to 0 days. Statistical test by Tukey’s HSD test.

The results for accelerated tests of the stability of antioxidative and neuroprotective activities of MMP found that throughout 8 weeks there were raised IC50 values of FRAP and inhibition of DPPH, AChE, MAO, MAO-A, and GABA-T in storage at 2, 4, and 8 weeks when compared to 0 days with significantly differences. We confirm that high temperature has an effect on reducing the shelf life of bioactivities in both real-time and accelerated tests, as shown in Figure 4.

### 3.4. MMP Powder Characteristics

Throughout six months, MMP powder experiments exhibited quite stable pH values in both the real-time and accelerated tests. Shelf life ranges were 4.52–4.53 and 4.52–4.57, respectively. The moisture in the real-time tests decreased from 6.25% to 3.58% and in the accelerated test decreased from 2.99% to 1.94%. The color (L*) indicates that lightness decreased from 44.44 to 27.85 and 39.27 to 34.59 in real-time and accelerated shelf-life tests, respectively. The color (a*) is the red/green coordinate; the red color decreased from 21.69 to 4.69 and from 23.81 to 18.93 in real-time and accelerated shelf-life tests, respectively. The color (b*) is the yellow/blue coordinate; the yellow color decreased from 7.53 to 3.13 and from 9.03 to 4.85 in real-time and accelerated shelf-life tests, respectively. % Brix of MMP increased from 2.90 to 4.50 and from 2.80 to 4.20 in real-time and accelerated shelf-life tests, respectively, as shown in Table 4.

### 3.5. Nutritional Content of a Mung Bean + Mulberry Fruit (1:3) Freeze-Dried Powder (MMP)

MMP contains 381 kcal of calories (including dietary fiber) per 100 g, with 0.73 g of total fat, 0.34 g of saturated fat, no detected cholesterol, 13.6 g of protein, 80.1 g of carbohydrates (including dietary fiber), 10.8 g of dietary fiber, 6.99 g of insoluble fiber, 3.82 g of soluble fiber, 42.2 g of total sugars, and 2.97 mg of sodium, as shown in Table 5.

## 4. Discussion

This study suggests that mung bean and mulberry fruit mix at an appropriate ratio (herein at ratio 1:3 (g/g)) could be used as a functional neuroprotective ingredient. The analysis of neuroprotective parameter IC_50_ values revealed an effect on antioxidant DPPH inhibition and FRAP of 5.652 mg/mL and 5.271 mg/mL, respectively, and against the activities of AChE, MAO, MAO-A, and GABA-T at 1.484 mg/mL, 4.575 mg/mL, 5.109 mg/mL, and 3.380 mg/mL, respectively. Interestingly, the results show a combination index (CI) < 1, suggesting a synergistic effect to inhibit the AChE enzyme. The cholinergic pathway involved in cognitive deficit and Alzheimer’s disease (AD) is the target of activity of the AChE enzyme that plays a role in breaking down or hydrolyzing acetylcholine, raising acetylcholine levels in the brain and resulting in improved cognitive functions in AD [33]. Additionally, a previous study of the CI values of mulberry fruits and mulberry leaves demonstrated that synergism inhibits DPPH and ABTS free radical scavenging formation, while being antagonistic to inflammation (Cyclooxygenase-II, COX-II) [34]. Thus, this study is the first to discover the CI values of mung bean and mulberry fruit mix, thereby improving knowledge of neuro-parameters.

MAO type A and B inhibitors are widely used in the treatment of psychiatric and neurological disorders [23]. Similarly, a study on healthy, working-age individuals who consumed one or two servings of anthocyanin-rich mulberry milk daily containing 34.30 ± 0.74 mg cyanidin-3-glucoside (C3G) per liter or 6.17 mg per serving suggested potential benefits for mental health, possibly through the suppression of MAO-A activity [35]. Additionally, Anthaplex soup, at a dosage of 2 g per serving and containing approximately 1.608 ± 0.653 mg C3G/g total anthocyanins has been shown to enhance cognitive function, improve working memory, and suppress AChE activity [36]. Mung beans and mulberry fruit, which provide approximately 2.869 ± 0.003 mg C3G/g per sample of total anthocyanins, may also have the potential to promote mental health, enhance cognitive function, improve working memory, and suppress AChE activity when consumed in amounts exceeding 3 g per day. Mung beans contain various neuroprotective polyphenols that could be a healthy food for AD prevention in AD animal models. It was hypothesized that vitexin, isovitexin, and ferulic acid are the major bioactive compounds for mung bean-mediated neuroprotection [37].

Mung beans and mulberry fruits are important for developing neuroprotective supplements, and this study indicates that the shelf-life stability of mung bean–mulberry powder (e.g., its phytochemical contents, antioxidants, and neuroprotective activities) stabilized for six months when stored at 4 °C better than when stored at room temperature. Thus, high temperature affects its phytochemical contents and bioactivity changes. A previous study showed that mulberry fruits contain TPC at 5.19 mg GAE/g fruit and TAC at 6.67 mg/g fruit, with DPPH inhibition at EC_50_ = 232.25 μg/mL [16]. A study of 12 mulberry genotypes showed various contents of anthocyanin between 0.51 and 28.61 mg/g DW [38], indicating that the difference in plant cultivars harvested each season is the factor resulting in different phytochemical contents. We used an MMP (mulberries Chaing Mai 60 species, harvested between 2022 and 2023 in Thailand) still stable in TPC, TAC, and GABA prolonged to six months, suggesting storage at ≤4 °C. GABA content in the soaked mung beans ranged from 1.59 to 32.33 mg/100 g DW [39]. Mung bean extract has a GABA content as high as 57.73 ± 1.28 mg/g DW [40], whereas the unextracted MMP contains only 2.151 ± 0.047 mg/g DW. Furthermore, the GABA content of MMP decreases significantly by 0.900 ± 0.029 mg/g DW after being stored for 6 months at room temperature, highlighting the difference in GABA levels between the extracted form and the raw plant material. However, we suggest that high temperature is the main factor affecting the phytochemical contents and bioactivities of MMP, thus the appropriate storage was at ≤4 °C and avoiding light to extend shelf life.

Phytonutrients of mung beans are excellent sources of protein, dietary fiber, minerals, vitamins, and significant amounts of bioactive compounds, including polyphenols, polysaccharides, and peptides [41]. The resultant LC-MS/MS found that MMP containing the major bioactive compounds vitexin and iso-vitexin from mung beans may have an effect on hyperglycemic activity and gut microbiota in weight modulation [42]. There has been identification of the amino acid composition of MMP (L-aspartic acid, D-phenylalanine, D-tryptophan, o-tyrosine, isoleucine, and L-threonine) while a previous study showed that the phenylalanine content of mung beans is greater than soy protein and other plant-based protein concentrates [10,12]. Phenylalanine is an essential amino acid which can improve performance-associated cognitive outcomes in adults with phenylketonuria [43]. Moreover, the result of phytochemical identification of MMP found that the dominant bioactive compounds from mulberry are cyanidin 3-rutinoside, rutin, quercetin 7-glucoside, cyanidin 3-glucoside (C3G), cyanidin 3-rutinoside (C3R), pel-argonidin 3-glucoside (pel-3-G), and delphinidin 3-rutinoside (D3R) [44,45,46], as show in the Supplementary Material.

Natural dietary fibers include insoluble and soluble fibers. Insoluble fibers are found in water (e.g., cellulose, hemicellulose; whole grains, and lignin in nuts and seeds) and are mostly derived from the outer skin of fruits and grains. Generally, they are not fermented by bacteria in the colon, resulting in them forming the bulk of feces and promoting laxation. While soluble fibers are derived from the inner flesh of plants, e.g., pectin, gums, and mucilage, they can form a viscous gel and can undergo fermentation by bacteria in the colon into gases and by-products such as short-chain fatty acids. They alter blood glucose and cholesterol concentrations [47]. We found that MMP contains both types of dietary fiber, with 6.99 g of insoluble fibers and 3.82 g of soluble fibers. A previous study demonstrated that daily consumption of dietary fiber can help prevent the symptoms of depression, anxiety, and stress [48,49], which are characteristic psychological states of negative mood (e.g., sad, fear, anxious, annoyed, or depressed) [50]. The American Heart Association recommends a total dietary fiber intake of 25–30 g/day, which may decrease the risk of obesity [51]. Thereby, the data indicate the possibility of MMP as a functional supplement that contains active phytonutrients, e.g., polyphenols, anthocyanins, and GABA, and exhibits neuroprotective effects along with containing macronutrients, e.g., high protein and dietary fiber, while also no cholesterol. Moreover, the data regarding shelf life could be applied in the food industry.

The underlying mechanism of the neuroprotective activity of combined mung bean and mulberry powder (MMP) first is antioxidant defense. It shows DPPH inhibition and FRAP that may relate to the reduction of reactive oxygen species (ROS) along with apoptosis (reduced caspases-8 marker) [52], and may improve oxidative stress status in the hippocampus, which is a brain region primarily associated with learning, memory, and emotion, by elevating of antioxidant enzyme activity such as superoxide dismutase (SOD), catalase (CAT), and glutathione peroxidase (GSH-Px) [53]. Second is cholinergic effect; it may act as an AChEI (acetylcholinesterase inhibitor), resulting in the reduced breakdown and accumulation of acetylcholine (ACh) as a major excitatory neurotransmitter in the brain, with cholinergic projections extending from the basal forebrain to the cerebral cortex and hippocampus and supporting cognitive function [54,55]. Third is that they act as MAOIs (monoamine oxidase inhibitors). Monoamine oxidase is an enzyme for degradation of amine neurotransmitters (serotonin, dopamine, norepinephrine, and epinephrine), thus the subsequent effects of MAOIs resulting in increasing monoamine concentration at synapses affect treatment of depression and neurological and psychiatric disorders [31]. Fourth is the GABAergic effect, as MMP affects GABA-T inhibition, affecting the accumulation of GABA, which is a major inhibitory neurotransmitter in the human nervous system, which improves the symptoms of stress, anxiety, and depression [32]. Fifth is the Gut–Brain Axis (GBA) effect, because MMP contains both soluble and insoluble fibers as the energy source for balancing the gut microbiota and subsequent greatly affects the bidirectional interaction between the brain and the gut through synchronized neuroendocrine mechanisms such as balancing the levels of stress hormones via the Hypothalamic–Pituitary–Adrenal (HPA) Axis, immunological mechanisms such as balancing systemic inflammation by gut dysbiosis defense [6,7], and neurological mechanisms via short-chain fatty acids (SCFAs). The postbiotic-producing gut microbiota play a role as a neuroactive compound involved in helping with mental health [56]. Possibly the five mechanisms mentioned above indicate that MMP provides initial data for its potential as a neuroprotection supplement. The in vivo study described mulberry fruit (containing cyanidin-3-glucoside, gallic acid, and quercetin-3-O-rutinoside) that are involved in enhancing cholinergic function and antioxidant enzyme activity [53], while the major phenolic compounds in mung beans, e.g., vitexin and isovitexin, were found in a pharmacokinetic trial in a rat model and interestingly these compounds can cross the blood brain barrier (BBB) which is why these compounds were detected in the rat brain [57].

## 5. Conclusions

Mung bean and mulberry fruit mix (MMP) at a ratio of 1:3 (g/g) showed the synergistic effect of AChE inhibition, and its shelf life showed stability of phytochemical contents and antioxidant and neuroprotective activities, which remained stabilized more than 50% in both real-time and accelerated conditions for 6 months and 8 weeks, respectively. The temperature of suitable storage is 4 °C or lower, which showed IC50 values of FRAP and inhibited DPPH, AChE, MAO, MAO-A, and GABA-T in ranges of 4.43–6.69 mg/mL, 4.10–4.68 mg/mL, 5.18–5.90 mg/mL, 4.95–5.43 mg/mL, 5.93–6.42 mg/mL, and 5.05–5.53 mg/mL respectively. Therefore, we demonstrated that the shelf life of MMP was six months, which stabilized for its neuroprotective activities and could be applied in the food industry for use as a healthful plant-based supplement. However, we require further study to ensure the mechanism of MMP in clinical trials.

## Figures and Tables

**Table 1:** IC50 values of biological activities from screening the nine ratios of mung bean/mulberry for antioxidant and neuroprotective activities. Data are presented as means of IC50 ± SEM (in triplicate experiments).

Biological Activities Parameters	IC_50_ (mg/mL) of Nine Ratios of Mung Bean/Mulberry									Mung Bean Alone	Mulberry Alone
	1:1	1:2	1:3	1:4	1:5	2:1	3:1	4:1	5:1		
Antioxidant activities											
DPPH inhibition	9.426 ± 0.057 #	6.662 ± 0.054 *,#	5.652 ± 0.062 *,#	5.977 ± 0.020 *,#	6.369 ± 0.017 *,#	8.043 ± 0.020 *	7.038 ± 0.023 *,#	7.470 ± 0.018 *,#	10.037 ± 0.094 *,#	8.892 ± 0.110	8.374 ± 0.095
FRAP	9.229 ± 0.111	7.143 ± 0.021 *,#	5.271 ± 0.051 *,#	5.512 ± 0.071 *,#	6.210 ± 0.039 *,#	8.526 ± 0.128	7.365 ± 0.149 *,#	7.468 ± 0.142 *,#	10.928 ± 0.093 *,#	8.865 ± 0.058	8.573 ± 0.026
Neuroprotective activities											
AChE inhibition	2.971 ± 0.016 *	1.909 ± 0.012 *,#	1.484 ± 0.005 *,#	1.709 ± 0.007 *,#	1.834 ± 0.005 *,#	2.373 ± 0.003 *	2.102 ± 0.005 *	2.244 ± 0.004 *	3.172 ± 0.012 *,#	4.952 ± 0.170 #	2.528 ± 0.013 *
MAO inhibition	8.000 ± 0.021 #	5.509 ± 0.051 *,#	4.575 ± 0.006 *,#	4.816 ± 0.023 *,#	5.164 ± 0.029 *,#	6.583 ± 0.013 *	5.967 ± 0.067 *,#	6.178 ± 0.019 *,#	8.805 ± 0.016 *,#	7.468 ± 0.096 #	6.831 ± 0.158 *
MAO-A inhibition	8.797 ± 0.111 *,#	5.968 ± 0.008 *,#	5.109 ± 0.006 *,#	5.414 ± 0.042 *,#	5.676 ± 0.051 *,#	6.941 ± 0.011 *,#	6.335 ± 0.030 *,#	6.572 ± 0.018 *,#	9.481 ± 0.050 *,#	7.880 ± 0.031 #	7.410 ± 0.038 *
GABA-T inhibition	5.723 ± 0.023 *,#	4.070 ± 0.016 *,#	3.380 ± 0.005 *,#	3.682 ± 0.008 *,#	3.857 ± 0.011 *,#	5.723 ± 0.028 *,#	4.316 ± 0.018 *,#	4.461 ± 0.018 *,#	6.106 ± 0.023 *,#	5.411 ± 0.012 #	5.162 ± 0.021 *

DPPH—1,1-diphenyl-2-picryl hydrazyl radicals; FRAP—Ferric Reducing Ability Power; AChE—acetylcholinesterase; MAO—monoamine oxidase; MAO-A—monoamine oxidase type A; GABA-T—gamma-aminobutyric acid transaminase; IC_50_—half maximal inhibitory concentration. *, #—*p* < 0.05 when compared to mung beans alone, and mulberries alone, respectively, Statistical tests by Tukey’s HSD test.

**Table 2:** The combination index (CI) values of a combined mung beans + mulberry fruit (1:3) and the dose reduction index (DRI) values of mung beans and mulberry fruit.

Parameters IC50 (mg/mL)	Mung Bean Alone	Mulberry Alone	A Combined Mix of Mung Bean + Mulberry Fruit (1:3)	Combination Index (CI)	Dose Reduction Index (DRI)	
					Mung Bean Alone	Mulberry Fruit Alone
Antioxidant activities						
DPPH inhibition	8.892 ± 0.110	8.374 ± 0.095	5.652 ± 0.062	1.31 ± 0.030 (additive)	1.57 ± 0.061	1.48 ± 0.019
FRAP	8.865 ± 0.058	8.573 ± 0.026	5.271 ± 0.051	1.21 ± 0.018 (additive)	1.68 ± 0.024	1.63 ± 0.032
Neuroprotective activities						
AChE inhibition	4.952 ± 0.170	2.528 ± 0.013	1.484 ± 0.005	0.88 ± 0.016 (synergism)	3.33 ± 0.217	1.70 ± 0.007
MAO inhibition	7.468 ± 0.096	6.831 ± 0.158	4.575 ± 0.006	1.29 ± 0.032 (additive)	1.63 ± 0.033	1.49 ± 0.058
MAO-A inhibition	7.880 ± 0.031	7.410 ± 0.038	5.109 ± 0.006	1.34 ± 0.011 (additive)	1.54 ± 0.013	1.45 ± 0.015
GABA-T inhibition	5.411 ± 0.012	5.162 ± 0.021	3.380 ± 0.005	1.28 ± 0.002 (additive)	1.60 ± 0.006	1.52 ± 0.007

DPPH—1,1-diphenyl-2-picryl hydrazyl radicals; FRAP—Ferric Reducing Ability Power; AChE—acetylcholinesterase; MAO—monoamine oxidase; MAO-A—monoamine oxidase type A; GABA-T—gamma-aminobutyric acid transaminase. Shelf life of the phytochemical contents of a mung bean + mulberry fruit (1:3) freeze-dried powder (MMP).

**Table 3:** Real-Time shelf-life stability of antioxidative and neuroprotective activities of MMP at room temperature and 4 °C for 0, 1, 3, and 6 months. Data are presented as mean ± SEM (in triplicate experiments).

Biological Activities Parameters	Real-Time Shelf Life of MMP (IC_50_ (mg/g dry Powder))						
	0-Day	1-Month		3-Month		6-Month	
	RT, 30 ± 2 °C	RT, 30 ± 2 °C	4 °C	RT, 30 ± 2 °C	4 °C	RT, 30 ± 2 °C	4 °C
Antioxidant activities							
DPPH inhibition	3.719 ± 0.015	4.411 ± 0.015 a	4.101 ± 0.014 a,b	9.645 ± 0.030 a	4.679 ± 0.015 a,b	9.640 ± 0.03 a	4.68 ± 0.02 a,b
FRAP	3.874 ± 0.026	4.788 ± 0.065 a	4.432 ± 0.030 a,b	9.945 ± 0.055 a	5.307 ± 0.035 a,b	10.11 ± 0.06 a	6.69 ± 0.05 a,b
Neuroprotective activities							
AChE inhibition	4.810 ± 0.020	5.372 ± 0.011 a	5.183 ± 0.004 a,b	5.747 ± 0.013 a	5.551 ± 0.009 a,b	6.21 ± 0.01 a	5.90 ± 0.03 a,b
MAO inhibition	4.545 ± 0.010	5.085 ± 0.016 a	4.958 ± 0.019 a	5.297 ± 0.011 a	5.198 ± 0.009 a	5.61 ± 0.01 a	5.43 ± 0.03 a
MAO-A inhibition	5.606 ± 0.0033	6.106 ± 0.007 a	5.939 ± 0.014 a,b	6.337 ± 0.002 a	6.094 ± 0.003 a,b	6.83 ± 0.01 a	6.42 ± 0.00 a,b
GABA-T inhibition	4.808 ± 0.004	5.195 ± 0.018 a	5.051 ± 0.004 a	5.447 ± 0.023 a	5.081 ± 0.022 a,b	5.76 ± 0.02 a	5.53 ± 0.01 a,b

DPPH—1,1-diphenyl-2-picryl hydrazyl radicals; FRAP—Ferric Reducing Ability Power; AChE—acetylcholinesterase; MAO—monoamine oxidase; MAO-A—monoamine oxidase type A; GABA-T—gamma-aminobutyric acid transaminase; MMP—mung bean + mulberry fruit (1:3) freeze-dried powder; IC_50_—half maximal inhibitory concentration; RT—room temperature; ^a, b^—*p* < 0.05 when compared at 0-day and at RT each time, respectively, Statistical test by Tukey’s HSD test.

**Table 4:** Powder characteristics of MMP. Data are presented as mean ± SEM (in triplicate experiments).

Powder Characteristics	Real-Time Shelf Life at 4 °C				Accelerated Shelf Life at 40 °C		
	0-Day	1-Month	3-Month	6-Month	2-Week	4-Week	8-Week
PH values	4.53 ± 0.012	4.52 ± 0.005	4.52 ± 0.006	4.53 ± 0.01	4.53 ± 0.010	4.52 ± 0.006	4.57 ± 0.006
% Moisture	6.25 ± 2.156	5.1 ± 0.73 a	4.47 ± 1.448 a	3.58 ± 0.300 a	2.99 ± 0.570	2.93 ± 0.880	1.94 ± 0.621 a
Color values							
L*	44.44 ± 6.637	36.48 ± 1.162 a	28.10 ± 2.157 a	27.85 ± 0.59 a	39.27 ± 5.499	35.07 ± 0.313 a	34.59 ± 1.187 a
a*	21.69 ± 0.446	22.12 ± 2.219	8.98 ± 2.577 a	4.69 ± 0.92 a	23.81 ± 2.203	21.81 ± 0.242 a	18.93 ± 0.238 a
b*	7.53 ± 0.488	8.23 ± 2.903 a	3.29 ± 0.62 a	3.13 ± 0.507 a	9.03 ± 0.602 a	7.19 ± 1.278 a	4.85 ± 0.096 a
% Brix	2.90 ± 0.058	3.16 ± 0.15 a	3.42 ± 0.153 a	4.50 ± 0.10 a	2.80 ± 0.173	3.40 ± 0.231 a	4.20 ± 0.058 a

L*—indicates lightness; a*—the red/green coordinate; b*—the yellow/blue coordinate; ^a^—*p* < 0.05 when compared to 0 days. Statistical tests by Tukey’s HSD test.

**Table 5:** Nutritional facts for mung bean + mulberry fruit (1:3) freeze-dried powder (MMP).

Parameters	100 g of MMP	Method
Calories (including dietary fiber) (kcal)	381	Method of analysis for nutrition labeling, 1993, p. 106
Total fat (g)	0.73	In-house method STM No.03-010 based on AOAC (2023) 996.06
Saturated fat (g)	0.34	
Cholesterol (mg)	Not Detected	In-house method STM No.03-027 based on Journal of AOAC International, Vol. 76, No. 4, 1993, p. 902–906
Protein (g)	13.6	In-house method STM No.03-017 based on AOAC (2023) 981.10
Carbohydrate (including dietary fiber) (g)	80.1	Method of analysis for nutrition labeling, 1993, p. 106
Dietary fiber (g)	10.8	In-house method STM No.03-008 based on AOAC (2019) 985.29
Insoluble fiber (g) Soluble fiber (g)	6.99 3.82	Based on AOAC (2023), 991.42 Based on AOAC (2023), 993.19
Total sugars (g)	42.2	In-house method STM No.03-025 based on Journal of AOAC International, Vol. 75, No. 3, 1992, p. 443–464
Sodium (mg)	2.97	In-house method STM No.05-013 based on AOAC (2023) 984.27

## Data Availability

The original contributions represented in the study are included in the article/Supplementary Materials. Further inquiries can be directed to the corresponding author.

## Supplementary Materials

The following supporting information can be downloaded at https://www.mdpi.com/article/10.3390/foods14060993/s1, Table S1. Phytochemical profiles in a mung bean and mulberry fruit powder mix by Liquid Chromatography–Mass Spectrometry (LC-MS/MS).
