# Supplementary material for: A Novel Combined Mung Bean and Mulberry Powder: Combination Index and Shelf Life of Total Phenolic, Anthocyanin, and GABA Contents and Neuroprotective Activity
Source: Foods. 2025 Mar 14;14(6):993. doi: 10.3390/foods14060993 (PMC11941386; doi:10.3390/foods14060993)
Supplement: Supplementary file 1 [file foods-14-00993-s001.zip › foods-3463911-supplementary.pdf]

Supplementary material 1

Table S1. Phytochemical profiles in a Mung bean mix Mulberry fruit powder by Liquid Chromatography–Mass Spectrometry (LC-MS/MS).

No.	Identification	MSMS Retention time (RT)	[M − H] (*m/z*)	MS/MS Fragment Ions (*m/z*)
Negative mode				
1	L-Aspartic acid	3.09	132.0228	88.0407, 62.7278
2	Gluconic acid	3.291	195.0403	159.0355, 129.0188, 75.0081
3	cis-Ferulic acid	3.383	193.0249	129.0146, 59.0158
4	Scopoletin	3.463	191.0455	127.0339, 78.9589
5	D-Phenylalanine	7.303	164.0616	103.058
6	D-Tryptophan	8.753	203.0699	116.0504, 74.0262
7	Epicatechin 4'-glucuronide	8.701	465.081	356.0589, 285.0405, 241.0515, 166.0256, 112.9773, 59.0139
8	Cyanidin 3-rutinoside	8.785	593.1216	549.2584, 482.4115, 327.0168, 284.0325, 241.0568, 125.016
9	Luteolin 7-glucoside	10.393	447.1288	401.1482, 325.1675, 269.1012, 233.0624, 161.0463, 101.0247, 71.0144
10	Rutin	11.93	609.1178	550.6053, 465.0493, 343.0522, 300.0254, 178.9888, 113.5305
11	Isovitexin	12.273	431.0778	341.0653, 311.0548, 239.0706, 117.0335
12	Quercetin 7-glucoside	12.586	463.066	353.08675, 300.02826, 178.99898, 79.01875
**No.**	**Identification**	**Retention time (RT)**	**[M + H] (*m/z*)**	**MS/MS Fragment Ions (*m/z*)**
Positive mode				
1	o-Tyrosine	3.883	182.0813	165.0528, 91.0547
2	Isoleucine	5.599	132.1022	86.0966, 55.0183
3	L-Threonine	7.314	120.0807	77.03814
4	Cyanidin 3-glucoside	8.97	449.1081	287.05449, 213.05387, 137.02271, 81.03527
5	Cyanidin 3-rutinoside	8.78	595.1652	535.13678, 449.10374, 330.12153, 287.05465, 253.16415, 189.05575, 128.05998, 72.33481
6	Pelargonidin 3-glucoside	9.304	433.1133	338.14284, 271.0591, 121.0278
7	Delphinidin 3-rutinoside	11.941	611.1614	465.10363, 340.66066, 303.04985, 258.04311, 85.02733
8	Vitexin	12.289	433.1133	397.092, 313.0701, 283.0594, 165.0184, 68.9962
